# Trauma scoring systems for evaluating trauma center triage criteria in older adults: a retrospective cohort study

**DOI:** 10.1186/s12245-026-01315-w

**Published:** 2026-07-29

**Authors:** Małgorzata Sulej-Niemiec, Andrzej Kopta, Magdalena Żurowska-Wolak, Łukasz Kręglicki, Mirosław Szura

**Affiliations:** 1 Faculty of Health Sciences, Department of Emergency Medical Services, Jagiellonian University Medical College, Michałowskiego St. 12, 31-126, Krakow, Poland; 2 Faculty of Health Sciences, Clinic of Surgery, Jagiellonian University Medical College, Krakow, Poland

**Keywords:** Geriatric trauma, Trauma triage, Trauma scoring systems, TRISS, Injury severity, Undertriage

## Abstract

**Background:**

Standardized trauma assessment is essential for early clinical decision-making; however, it remains challenging in older adults because age-related physiological changes may obscure injury severity. Accurate triage is critical to ensure timely access to trauma centers (TCs). While triage decisions must rely on immediately available clinical findings, advanced trauma scoring systems are typically based on diagnostic data obtained later in the course of care. Although these tools are not suitable for real-time triage, they are established predictors of mortality and may provide an objective basis for evaluating TC qualification criteria. Because current national TC qualification criteria do not adequately account for age-specific factors, they may contribute to undertriage in older patients. This study aimed to retrospectively identify the trauma scoring system most suitable for evaluating TC qualification criteria in geriatric trauma patients.

**Methods:**

We conducted a retrospective cohort study of patients admitted to the emergency department of a single clinical hospital with a TC between 2017 and 2020. Among 155,320 ED admissions, 6,541 trauma patients aged ≥ 65 years were identified. Injury severity and physiological status were assessed using the coded Revised Trauma Score (RTSc), Injury Severity Score (ISS), and Trauma and Injury Severity Score (TRISS). A predefined subgroup of patients meeting national TC qualification criteria (TC group 65+) was analyzed separately. Predictive performance for in-hospital mortality was evaluated using receiver operating characteristic (ROC) curve analysis.

**Results:**

TRISS showed the highest discrimination for in-hospital mortality (AUC 0.888), followed by ISS (0.867) and RTSc (0.687). In *the TC group 65+*, the mean TRISS-predicted survival probability was 34.58%, whereas observed in-hospital survival was 48.72%. The estimated undertriage rate was 73.47%, suggesting that current national TC qualification criteria may perform inadequately in older trauma patients.

**Conclusion:**

Current national TC qualification criteria may contribute to undertriage among older patients, potentially delaying access to advanced trauma care. Among the evaluated scoring systems, TRISS demonstrated the highest discriminatory performance for in-hospital mortality and appears to be the most suitable retrospective reference standard for assessing TC qualification criteria in geriatric trauma patients.

## Introduction

Accurate assessment of injury severity is fundamental to trauma-related clinical decision-making, triage, and outcomes research. Trauma scoring systems convert anatomical and physiological injury patterns into numerical values, enabling standardized patient evaluation and comparison of treatment outcomes across institutions and settings. Since the introduction of the Abbreviated Injury Scale (AIS) more than five decades ago, numerous trauma scoring systems have been developed, reflecting both the continuing clinical need for reliable assessment tools and the limitations of existing methods [1].

Despite continuous refinement, standardizing trauma severity assessment remains challenging because physiological responses to injury are complex and heterogeneous, particularly in patients with multiple traumatic injuries. These challenges are especially pronounced in geriatric patients, in whom the impact of trauma is not merely additive, making conventional severity assessments less reliable [2, 3].

Trauma scoring systems serve different purposes in emergency care. Simpler tools are used in the prehospital setting to support rapid triage and trauma center referral, where decisions must be based on parameters immediately available at the scene or on initial examination. In contrast, more complex scoring systems often rely on diagnostic information obtained later in the hospital course and are therefore used primarily for retrospective outcome assessment, benchmarking, and mortality prediction. Although these tools are valuable for evaluating trauma care and comparing outcomes between centers, most currently used scoring systems—whether anatomical, physiological, or combined—do not adequately account for age-specific factors, which may reduce their accuracy in older trauma patients [2, 4–12].

Assessing injury severity in geriatric patients is particularly challenging because aging is associated with reduced physiological reserve, multimorbidity, polypharmacy, and other factors affecting baseline health status, including frailty and malnutrition [13–16]. These factors can blunt the physiological response to trauma, impair compensation for hemorrhage and ischemia, increase susceptibility to complications, and ultimately contribute to higher post-trauma mortality [17–24]. For example, the ability to restore hemodynamic balance through autotransfusion is diminished in older adults, and age-related physiological changes may mask the severity of injury despite substantial trauma burden [24, 25]. As a result, injury severity may be underestimated in this population, directly affecting triage decisions and referral to specialized trauma care.

Accurate trauma assessment in older adults is essential to ensure timely access to specialized trauma care. In Poland, trauma center (TC) qualification criteria are defined in the 2010 Regulation of the Minister of Health (Table 1).

**Table 1:** National trauma center qualification criteria

No.	Anatomical injuries	Physiological parameters abnormalities
1.	Penetrating injuries to the head or torso, or blunt trauma with signs of internal organ injury to the head, chest, or abdomen	Systolic blood pressure ≤ 80 mmHg
2.	Amputation of an extremity above the knee or elbow	Pulse rate ≥ 120 beats/min
3.	Extensive crush injury to the extremities	Respiratory rate < 10 or > 29 per minute
4.	Spinal cord injury	Glasgow Coma Scale score ≤ 8
5.	Fracture of an extremity with vascular and nerve injury	Arterial blood saturation ≤ 90%
6.	Fracture of two or more proximal long bones of the extremities or pelvis	

The decision to refer a patient to a trauma center requires the simultaneous presence of at least two anatomical injuries and at least two abnormalities in physiological parameters [26]. Although these criteria integrate anatomical and physiological indicators, they are applied uniformly to all adult patients and do not account for age-related physiological differences.

In geriatric trauma patients, an atypical clinical presentation, often characterized by minimal physiological derangement and subtle anatomical signs despite severe injury, may lead to inappropriate triage and inadequate referral to a trauma center, consistent with the high rates of undertriage reported in recent studies [27–32, 51].

However, appropriate evaluation of TC qualification criteria in geriatric patients requires the identification of a suitable assessment tool that could serve as an objective reference standard. Therefore, this study focused on identifying such a tool.

### Objective

To retrospectively analyze data from geriatric trauma patients to identify the trauma scoring system best suited for evaluating trauma center triage and qualification criteria.

## Methods

This retrospective study analyzed the medical records of patients admitted to the Emergency Department (ED) of a single clinical hospital with a trauma center (TC) between 1 January 2017 and 31 December 2020. Among the 155,320 patients treated in the ED (overall study population), 6,541 trauma patients aged 65 years and older were identified based on ICD-10 diagnostic codes (trauma group 65+). Only complete cases were included in the final analysis, and no imputation of missing data was performed.

For each patient, trauma was assessed using the most widely used scoring systems representing the three main categories of trauma assessment: the coded Revised Trauma Score (RTSc) as a physiological scale, the Injury Severity Score (ISS) as an anatomical scale, and the Trauma and Injury Severity Score (TRISS) as a combined anatomical–physiological scale.

**Fig. 1:**
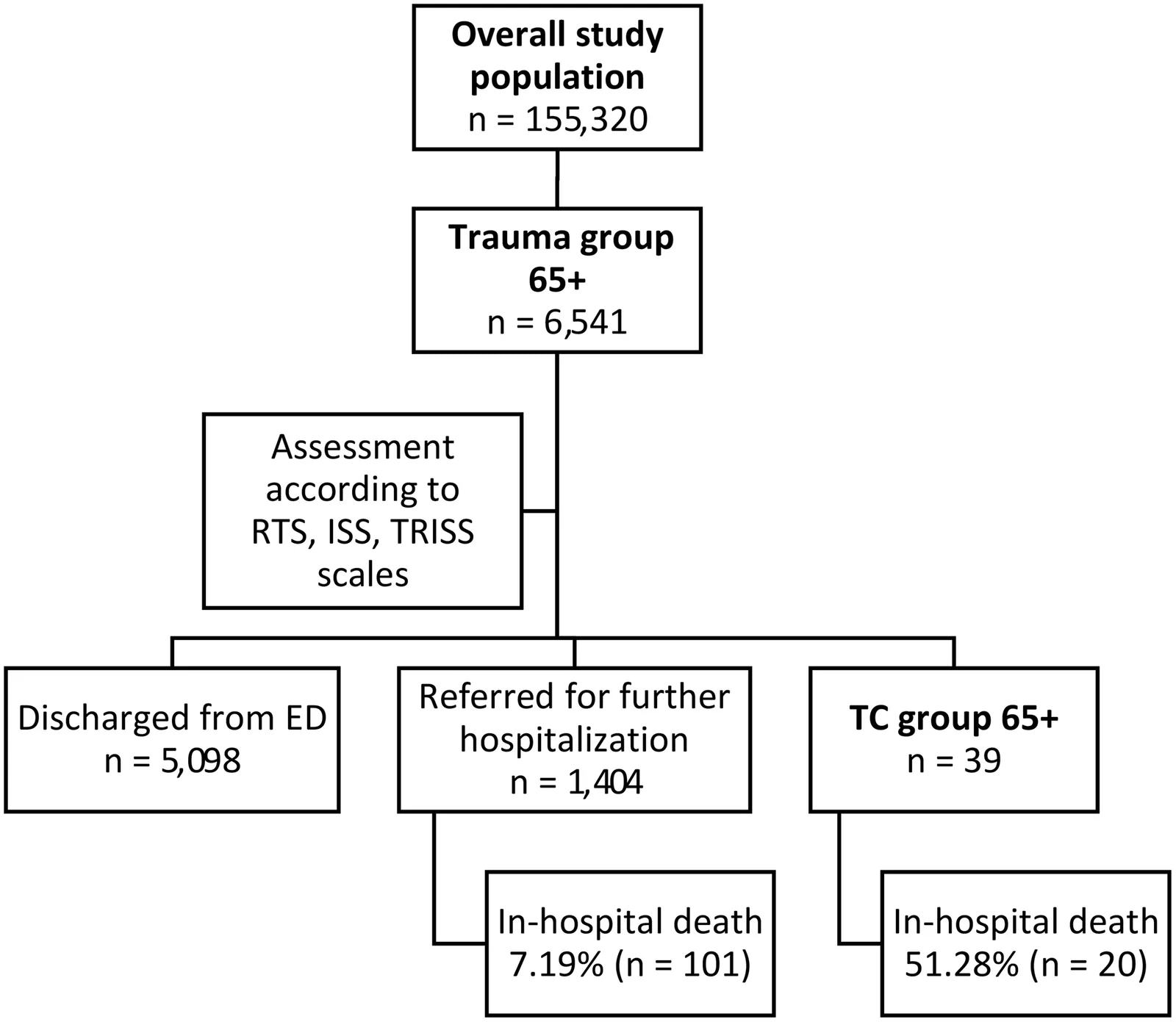
Flowchart of patient selection for the study

A predefined subgroup of patients meeting the national TC qualification criteria was also identified (*TC group 65+*; *n* = 39) (Fig. 1).

### Statistical analysis

Categorical variables were presented as counts and percentages. Continuous variables, including RTSc, ISS, and TRISS, were assessed for normality using the Shapiro–Wilk test and by visual inspection of histograms. As none of the variables were normally distributed, they were primarily described using medians and interquartile ranges (IQRs). Means and standard deviations (SDs) were also reported to provide descriptive completeness and to enable comparison with previous studies.

The ability of RTSc, ISS, and TRISS to predict in-hospital mortality in patients aged 65 years and older was evaluated using receiver operating characteristic (ROC) curve analysis. Discriminatory performance was quantified using the area under the ROC curve (AUC), with higher AUC values indicating better discrimination. The optimal cutoff value for each score was identified using the Youden index, which maximizes the sum of sensitivity and specificity. Sensitivity, specificity, positive predictive value (PPV), and negative predictive value (NPV) were calculated at the optimal cutoff point.

AUC values were compared pairwise using DeLong’s test for correlated ROC curves, using custom statistical procedures. Associations between categorical variables were assessed using the chi-square test or Fisher’s exact test, as appropriate. All statistical tests were two-sided, and p-values < 0.05 were considered statistically significant. Analyses were conducted using Statistica software, version 13 (StatSoft Inc.).

#### The Trauma and Injury Severity Score (TRISS)

was developed by the American College of Surgeons as part of the Major Trauma Outcome Study (MTOS), based on research initiated in 1982 and published in 1990 [33, 34]. The aim was to create a tool for estimating survival probability in patients with multiple injuries and to develop a prognostic model that would enable standardization and comparison of treatment outcomes across centers [1]. Despite substantial changes in trauma systems and concerns regarding its contemporary predictive value [5, 6, 33–36], TRISS has stood the test of time, achieving “gold standard” status [35] and remaining widely used as a combined model [37].

**Fig. 2:**
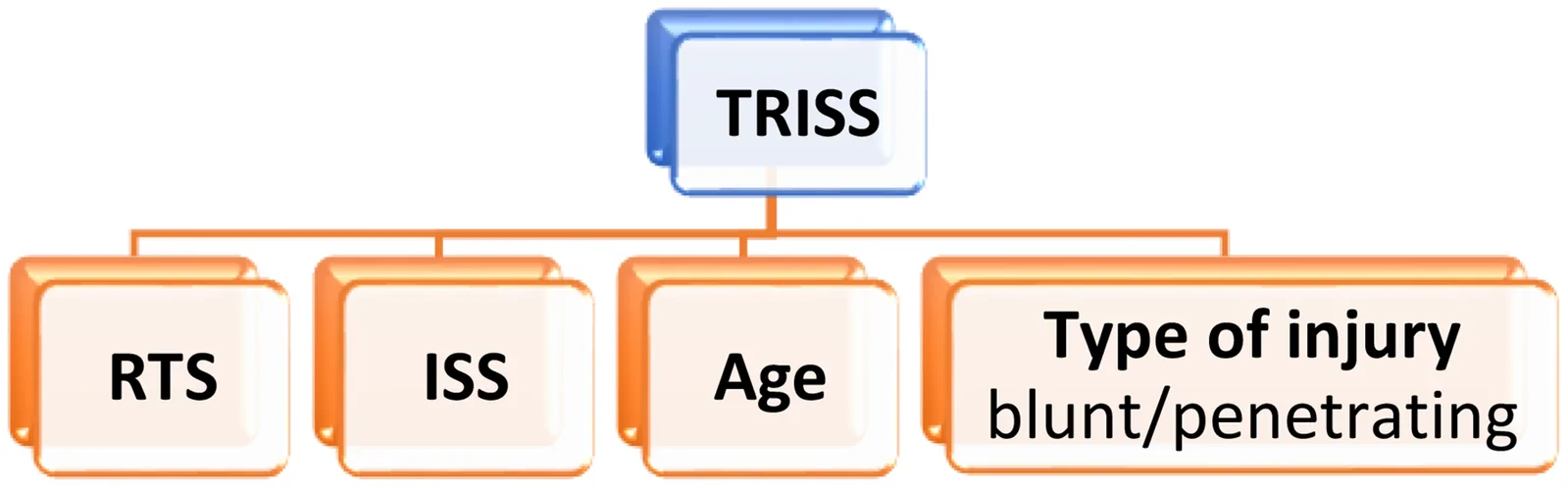
Components of the TRISS scale

TRISS predicts patient survival based on a combination of anatomical and physiological trauma scales. The components include the Injury Severity Score (ISS) and the coded version of the Revised Trauma Score (RTS). The model also differentiates between injury mechanisms (blunt vs. penetrating) [5] and incorporates an age variable with a cutoff of 55 years (≥ 55 = 1, < 55 = 0) [35]. The components of TRISS are shown in Fig. 2.

The TRISS model uses logistic regression to derive coefficients (β) that assign weights to each variable [35]. The probability of survival (Ps) is calculated using the following Eq. (38): **Ps** = 1/ (1 + e^− b^). In this equation, the coefficient b is calculated using separate formulas for blunt and penetrating injuries [33, 34, 38]:

$$\begin{aligned}\textbf{b}_\mathrm{blunt} &= -0.4499 + 0.8085\cdot{\mathrm{RTSc}}-0.0835\cdot{\mathrm{ISS}}\\ &\quad -1.7430\cdot{\text{age factor}}\end{aligned}$$

$$\begin{aligned} \textbf{b}_\mathrm{penetrating} &= -2.5355 + 0.9934\cdot{\mathrm{RTSc}}-0.0651\cdot{\mathrm{ISS}}\\&\quad -1.1360\cdot{\text{age factor}}. \end{aligned}$$

The resulting value represents the probability of survival (Ps), expressed as a percentage [1, 5, 6].

#### The Revised Trauma Score (RTS)

is a modification of the Trauma Score (TS), originally developed by Champion et al. in 1981, with the RTS model introduced in 1989 to improve predictive performance [39, 40]. RTS is a widely used physiological scale, applied either alone or in combination with other scales [1], and is based on three routinely assessed parameters: systolic blood pressure (SBP), respiratory rate (RR), and Glasgow Coma Scale (GCS) score [35]. Each parameter is assigned a coded value from 0 to 4, and the three coded values are summed to yield a total score ranging from 0 to 12 (Table 2) [1].

RTS is available in two forms: an unweighted triage version (range, 0–12), in which an RTS value < 11 may suggest the need for transport to a TC [39], and a coded, weighted version (RTSc), used mainly for outcome assessment. In RTSc, weighted coefficients are applied to each coded parameter: RTSc = 0.9368 × GCSc + 0.7326 × SBPc + 0.2908 × RRc [1]. RTSc ranges from 0 to 7.8408, with lower values indicating a poorer prognosis; an RTSc < 4 has been suggested as indicating the need for immediate TC care [8, 9].

An advantage of RTSc is the high weighting assigned to GCS, reflecting the impact of traumatic brain injury on outcomes [2, 40], although RTS has limitations that are largely related to GCS assessment [36].

In this study, RTSc was calculated using SBP, RR, and GCS values recorded at ED admission and coded according to Table 2.

**Table 2:** Revised trauma score (RTS)

Coded value	SBP (mm Hg)	RR (/min)	GCS
4	> 89	10–29	13–15
3	76–89	> 29	9–12
2	50–75	6–9	6–8
1	1–49	1–5	4–5
0	0	0	3

#### The Injury Severity Score (ISS)

is an anatomical injury rating system based on the Abbreviated Injury Scale (AIS).

#### The AIS

was developed in response to the need to classify injury severity in traffic accidents. Since its initial publication in 1971, it has undergone continuous revision; the AIS Dictionary: The Abbreviated Injury Scale © 2015 Revision includes more than 2,000 terms for classifying injury severity according to anatomical region and tissue damage, and serves as a unique guide to injury severity classification. Each injury is assigned an AIS severity score ranging from 1 to 6, with an additional code of 9 for injuries that cannot be evaluated (Table 3). AIS is not routinely calculated at admission, because it typically requires a detailed review of diagnostic documentation, and it serves both as a score for individual injuries and as a core component of multiple-injury scoring systems [41].

**Table 3:** AIS injury severity indices

No.	AIS index	Injury severity description
1.	1	Minor
2.	2	Moderate
3.	3	Serious
4.	4	Severe
5.	5	Critical
6.	6	Maximal
7.	9	Unknown

#### The ISS

was introduced by Baker et al. in 1974 [35] to standardize the assessment of multiple injuries [7]. In the ISS, injuries are evaluated across six body regions: head or neck, face, chest, abdominal or pelvic contents, extremities or pelvic girdle, and external structures (1, 41). It should be noted that these regions do not exactly correspond to those defined in the AIS Dictionary. To calculate the ISS, the highest AIS severity score is selected from each of the three most severely injured body regions, then squared and summed. The general formula for ISS is as follows: ISS = (AIS₁)² + (AIS₂)² + (AIS₃)².

The ISS ranges from 1 to 75 and correlates with the risk of death. A maximum score of 75 can result from either three AIS 5 injuries or one AIS 6 injury and is considered potentially fatal [41]. ISS is widely used in clinical trials and is an integral part of assessment systems based on trauma registries [41]. Many trauma registries include hospitalized patients with an ISS ≥ 9 [14]. In addition, according to the recommendation of the ACS Committee on Trauma (ACS COT), an ISS ≥ 16 is considered indicative of severe injury and an indication for treatment in a TC [7]. ISS categorization is presented in Table 4 [8, 42]. Among the limitations of ISS, the most notable are its inability to account for multiple injuries within the same body region, which may lead to underestimation of the score [33], and its restriction to three body regions, which may result in other significant anatomical injuries being overlooked [8].

**Table 4:** Severity of injury according to ISS

ISS score	Multiple-injury severity description
< 9	Mild
9–15	Moderate
**16**–24	Severe
≥ **25**	Profound

## Results

The overall study population comprised 155,320 patients, including 77,225 women (49.72%) and 78,095 men (50.28%). Patients ranged in age from 1 to 102 years (mean 49 years; SD 21).

### Trauma group 65+

**Fig. 3:**
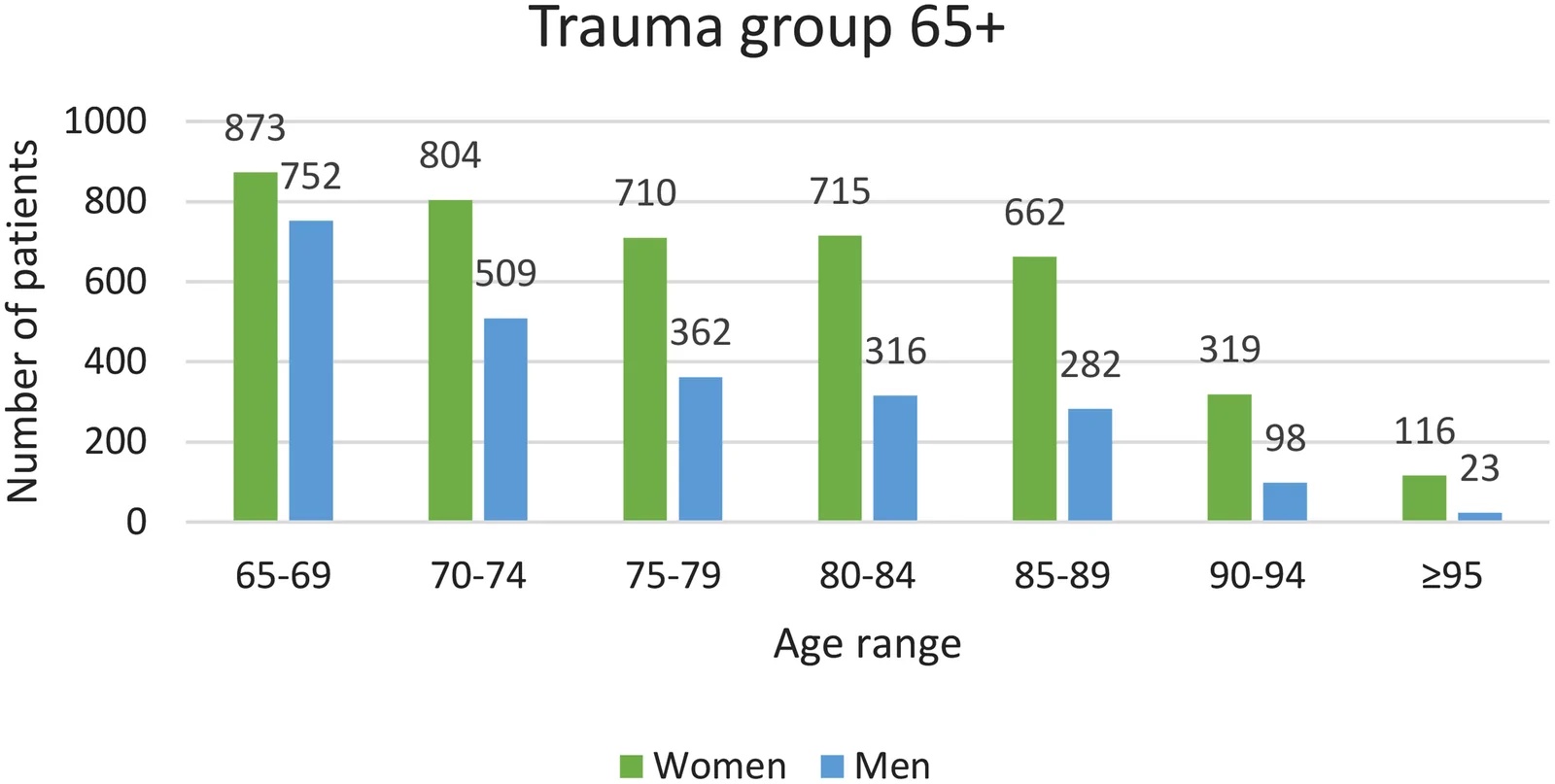
Distribution of *the trauma group 65 +* by age and sex

*The trauma group 65 +* consisted of 6,541 patients, including 4,199 women (64.20%) and 2,342 men (35.80%). Patients ranged in age from 65 to 102 years (mean 77 years; SD 8.48). The age and sex distribution of *the trauma group 65 +* is shown in Fig. 3.

The largest number of patients in *the trauma group 65 +* was observed in the 65–69-year age group, and women predominated across all age groups.

In *the trauma group 65+*, blunt trauma predominated, accounting for 6,508 patients (99.50%), whereas penetrating trauma was observed in 33 patients (0.50%). Based on the analysis of primary ICD-10 diagnoses, the most common injuries were head injuries (1,932 patients, 29.54%), hip and thigh injuries (803 patients, 12.28%), and elbow and forearm injuries (665 patients, 10.17%).

Descriptive statistics for RTSc, ISS, and TRISS are presented in Table 5. All analyzed scales deviated significantly from a normal distribution. Accordingly, medians and interquartile ranges are reported alongside means and standard deviations for descriptive completeness.

**Table 5:** Descriptive statistics for RTSc, ISS, and TRISS in *the trauma group 65+*

Type of scale	Trauma group 65 + *n* = 6,541			
	Minimum	Maximum	Mean (SD)	Median [IQR]
**RTSc**	1.47	7.84	7.80 (0.39)	7.84 [7.84–7.84]
**ISS**	1.00	66.00	3.47 (4.59)	1.00 [1.00–4.00]
**TRISS** %	0.42	98.31	97.17 (7.08)	98.31 [97.84–98.31]

The high median RTSc and TRISS values, together with the low median ISS, indicate that most patients presented with minor injuries and a high predicted probability of survival.

In the *trauma group 65+*, in-hospital mortality was 1.88% (*n* = 123), whereas survival was 98.12% (*n* = 6,418). A significant association was found between sex and in-hospital mortality, with higher mortality among men (χ² test, *p* < 0.001; Fisher’s exact test, *p* < 0.001).

**Fig. 4:**
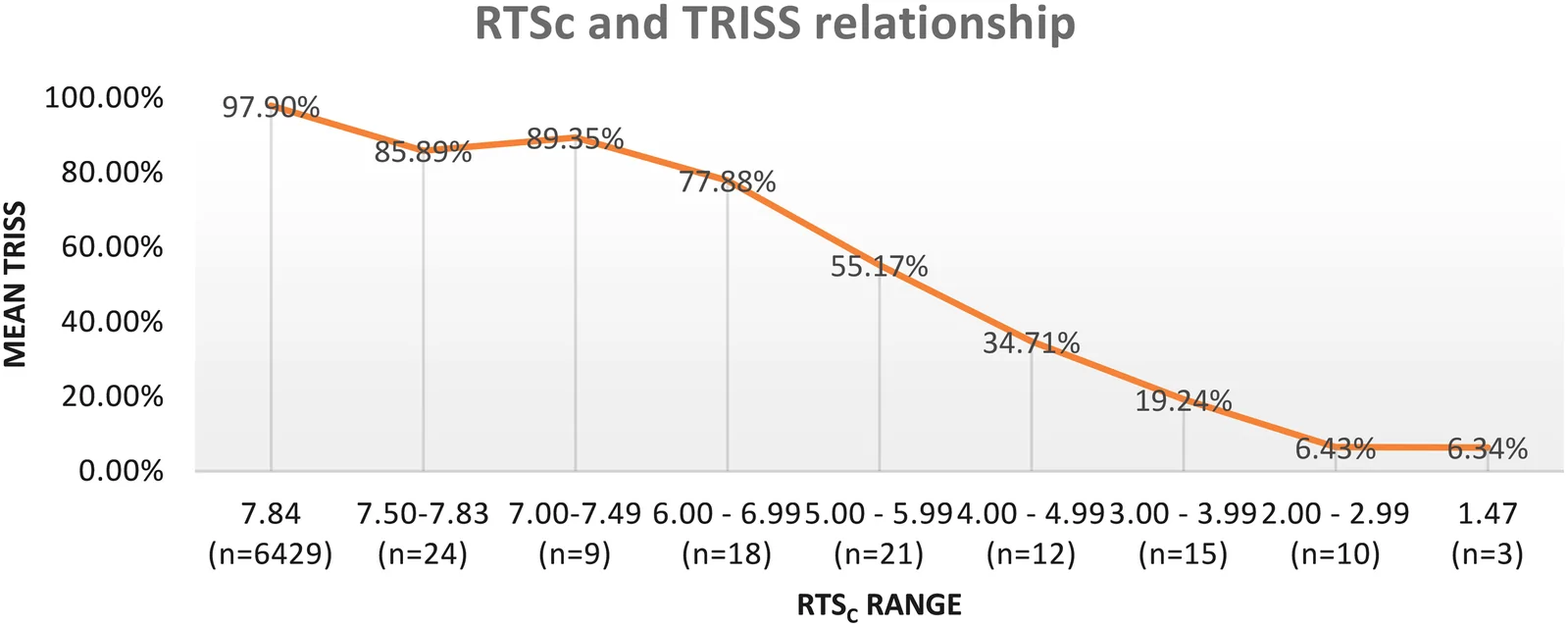
Relationship between RTSc and TRISS scores in *the trauma group 65+*

The relationship between trauma severity according to RTSc and the TRISS-predicted probability of survival in *the trauma group 65 +* is shown in Fig. 4.

In *the trauma group 65+*, patients with an RTSc < 4 accounted for 0.43% of the cohort (*n* = 28). Their mean TRISS-predicted probability of survival was 13.28% (SD 11.75). Among these patients, 39.29% (*n* = 11) survived hospitalization, with a mean TRISS-predicted probability of survival of 17.98% (SD 12.54).

**Fig. 5:**
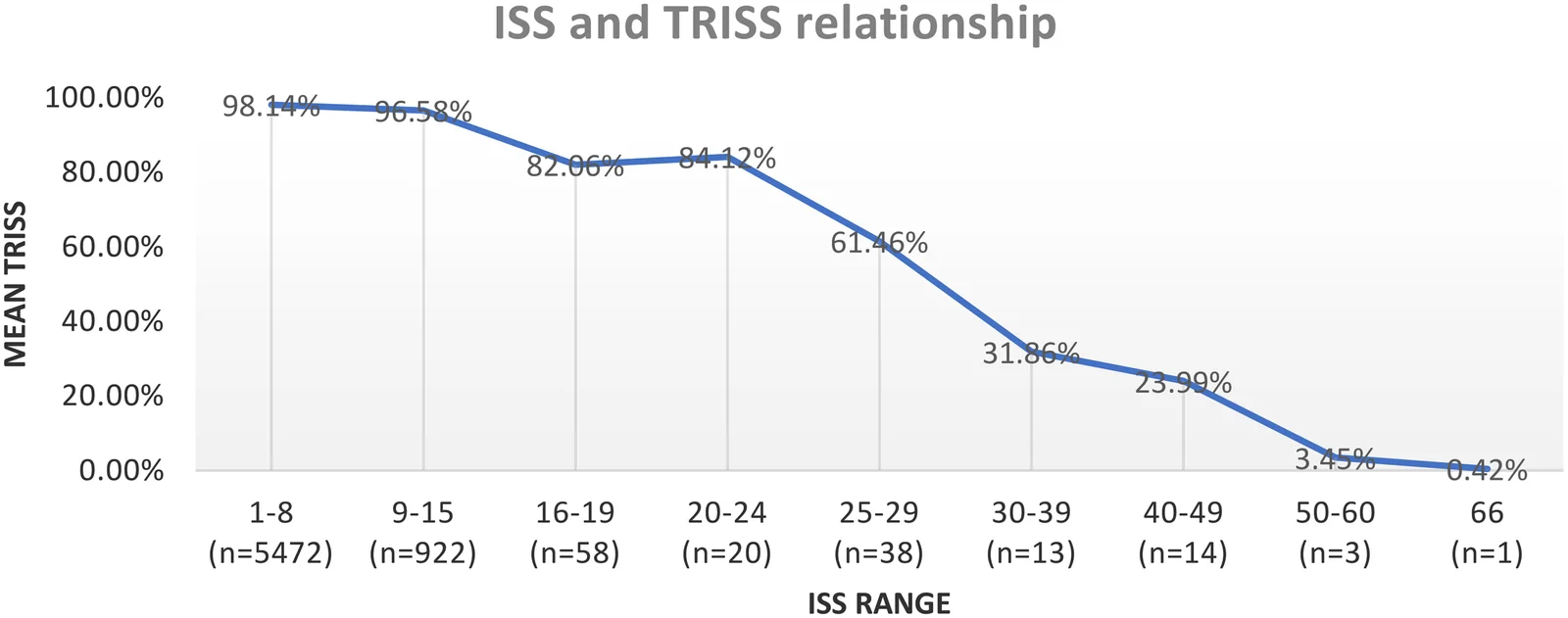
Relationship between ISS and TRISS scores in *the trauma group 65+*

The relationship between injury severity according to ISS and the TRISS-predicted probability of survival in *the trauma group 65 +* is shown in Fig. 5.

In *the trauma group 65+*, patients with an ISS ≥ 16 accounted for 2.25% of the cohort (*n* = 147). Their mean TRISS-predicted probability of survival was 64.89% (SD 32.77). Among these patients, 67.35% (*n* = 99) survived hospitalization, with a mean TRISS-predicted probability of survival of 75.29% (SD 27.31).

### TC group 65+

**Fig. 6:**
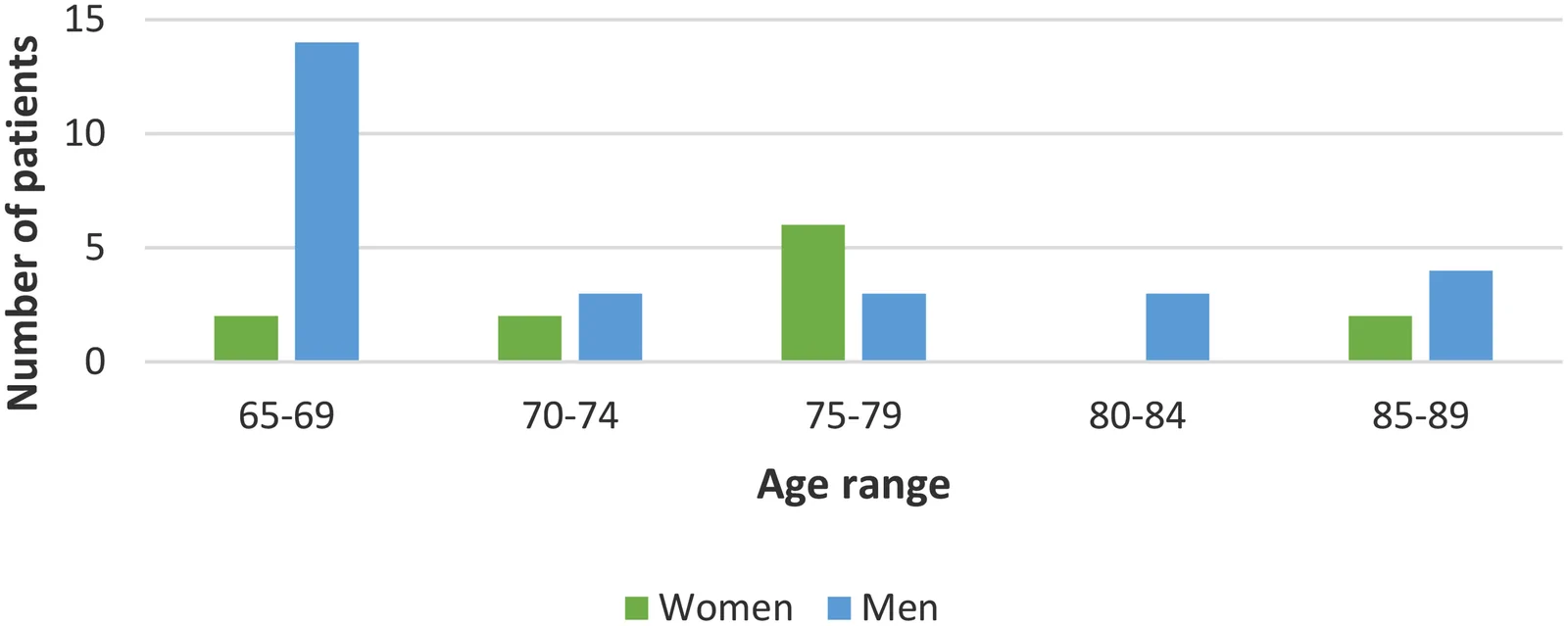
Age and sex distribution of *the TC group 65+*

*The TC group 65 +* consisted of 39 patients, including 12 women (30.77%) and 27 men (69.23%). Patients ranged in age from 65 to 88 years (mean 74 years; SD 7.13). This subgroup accounted for 0.60% of *the trauma group 65+*. The age and sex distribution of *the TC group 65 +* is shown in Fig. 6.

The largest number of patients in *the TC group 65 +* was observed in the 65–69-year age group, and men predominated in most age groups.

In *the TC group 65+*, all injuries were blunt (39 patients, 100%). Based on the analysis of primary ICD-10 diagnoses, the most common injuries were those involving multiple body regions (21 patients, 53.85%), chest injuries (6 patients, 15.38%), and head injuries (5 patients, 12.82%).

Descriptive statistics for RTSc, ISS, and TRISS in *the TC group 65 +* are presented in Table 6.

**Table 6:** Descriptive statistics for RTSc, ISS, and TRISS in *the TC group 65+*

Type of scale	TC group 65 + *n* = 39			
	Minimum	Maximum	Mean (SD)	Median [IQR]
**RTSc**	1.47	7.55	5.01 (2.02)	5.15 [3.51–7.11]
**ISS**	19.00	66.00	36.54 (11.17)	38.00 [26.00–43.00]
**TRISS** %	0.42	88.84	34.58 (32.21)	20.41 [5.41–60.62]

In *the TC group 65+*, in-hospital mortality was 51.28% (*n* = 20), whereas survival was 48.72% (*n* = 19). Fulfillment of TC criteria was strongly associated with in-hospital mortality (χ² test, *p* < 0.001; Fisher’s exact test, *p* < 0.001). Among survivors, the mean TRISS-predicted probability of survival was 47.35% (SD 32.39).

**Fig. 7:**
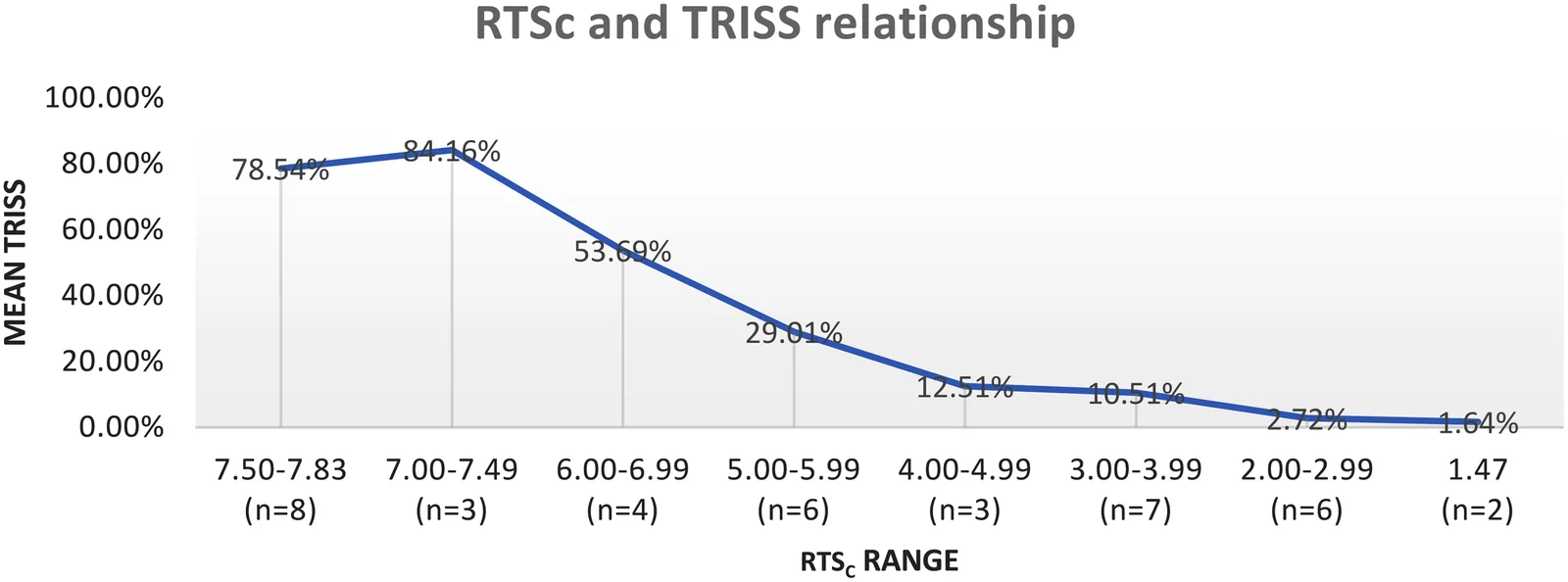
Relationship between RTSc and TRISS scores in *the TC group 65+*

The relationship between trauma severity according to RTSc and the TRISS-predicted probability of survival in *the TC group 65 +* is shown in Fig. 7.

In *the TC group 65+*, patients with an RTSc < 4 accounted for 38.46% of the group (15 patients). In this subgroup, the mean TRISS-predicted probability of survival was 6.21% (SD 5.79). Among patients with an RTSc < 4, 33.33% (5/15) survived hospitalization, with a mean TRISS-predicted probability of survival of 11.34% (SD 6.82).

**Fig. 8:**
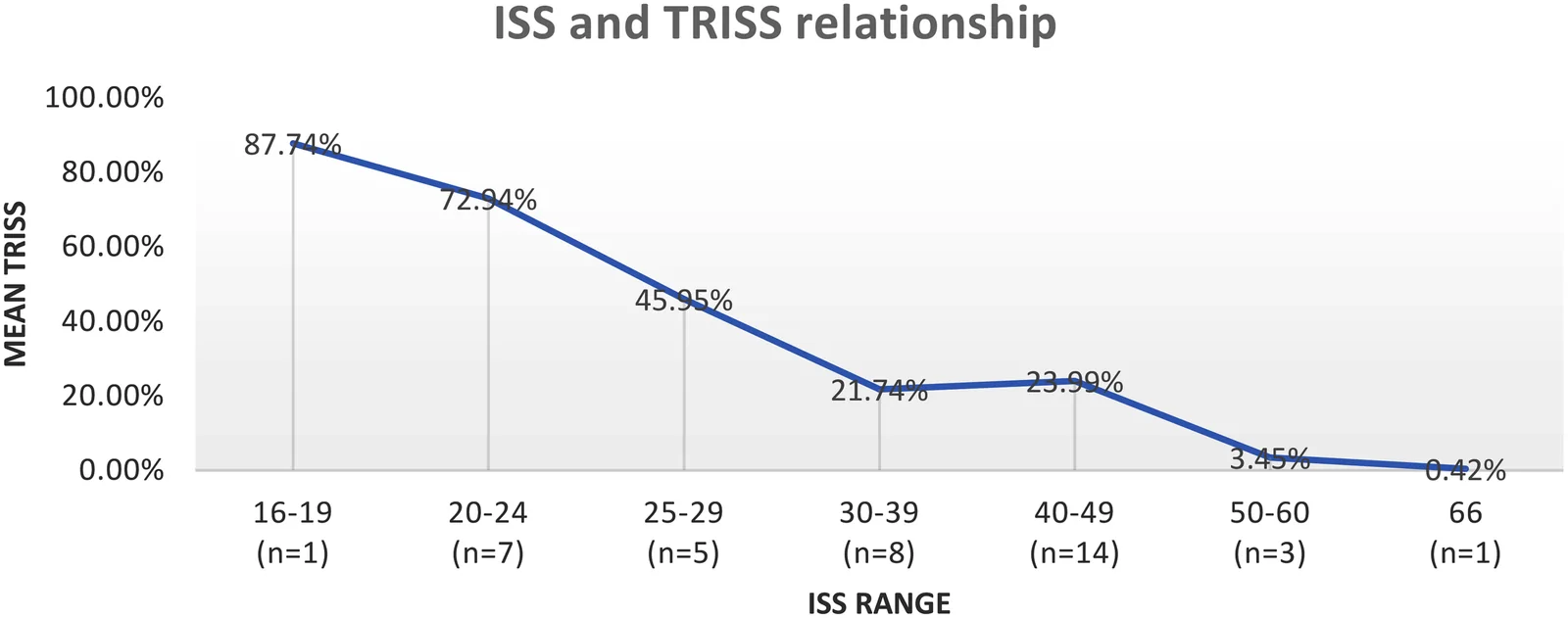
Relationship between ISS and TRISS scores in *the TC group 65+*

The relationship between injury severity according to ISS and the TRISS-predicted probability of survival in *the TC group 65 +* is shown in Fig. 8.

In *the TC group 65+*, all patients had an ISS ≥ 16 (39 patients, 100%). In this group, the mean TRISS-predicted probability of survival was 34.58% (SD 32.21). Among these patients, 48.72% (19/39) survived hospitalization, with a mean TRISS-predicted probability of survival of 47.35% (SD 32.39).

### Comparison of scales

**Fig. 9:**
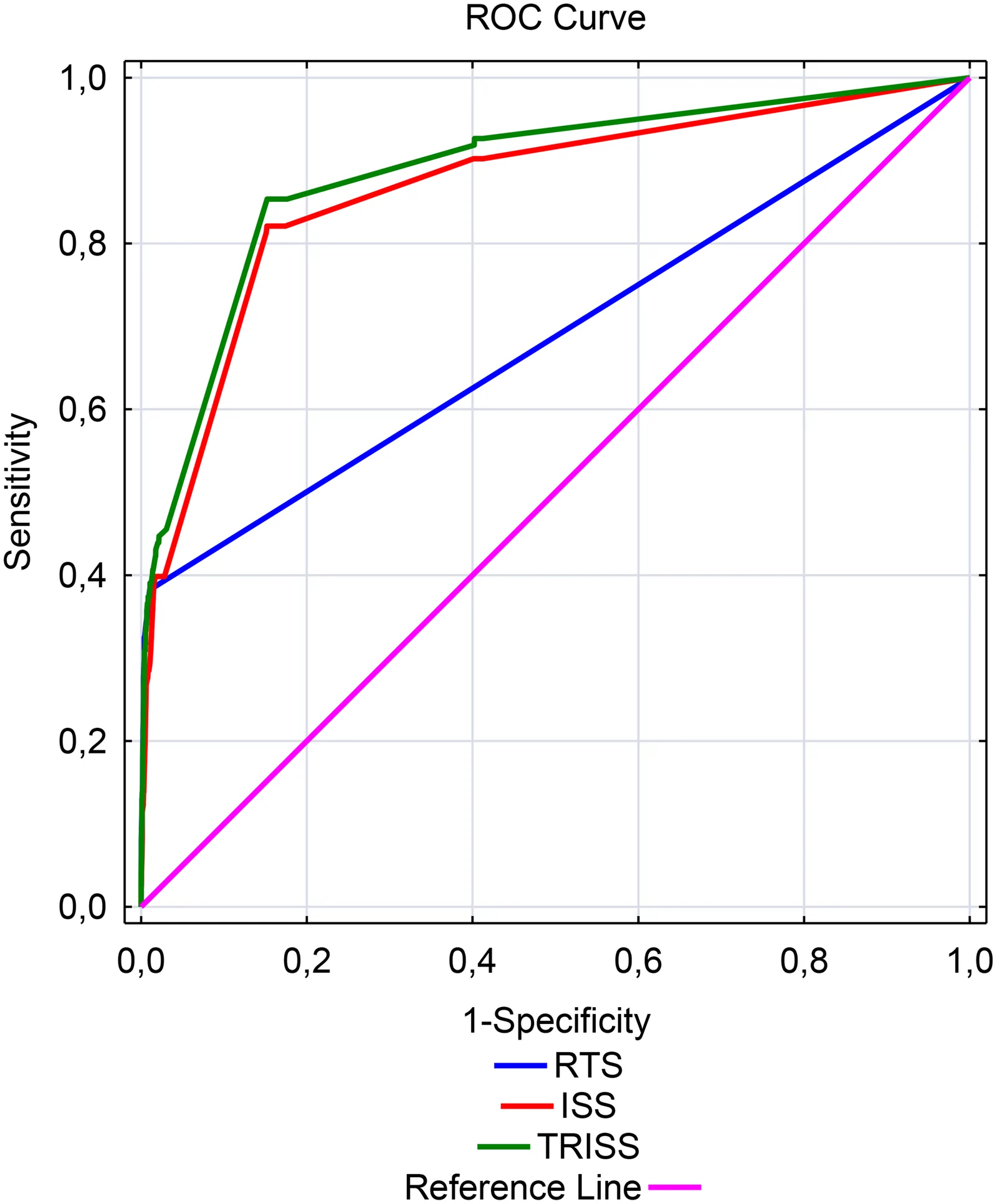
ROC curves for RTSc, ISS, and TRISS in predicting in-hospital mortality in patients 65+

The predictive performance of RTSc, ISS, and TRISS for in-hospital mortality in patients aged 65 years and older was compared using receiver operating characteristic (ROC) curve analysis (Fig. 9).

The superior performance of TRISS in predicting in-hospital mortality in this population was reflected by the largest area under the ROC curve (AUC-ROC). Detailed results of the ROC analysis are presented in Table 7.

**Table 7:** Comparison of RTSc, ISS, and TRISS in predicting in-hospital mortality

Scale	Cutoff	Sensitivity %	Specificity %	PPV %	NPV %	AUC (95% CI)	Youden Index	*p*-value
**RTSc**	≤ 7.55	38.20	99.00	41.96	98.82	0.687 (0.644–0.730)	0.372	< 0.001
**ISS**	≥ 8	82.10	84.80	9.39	99.60	0.867 (0.831–0.903)	0.669	< 0.001
**TRISS**	≤ 97%	85.40	84.70	9.71	99.67	0.888 (0.856–0.921)	0.701	< 0.001

ROC curve analysis showed that TRISS had the highest discriminatory ability for in-hospital mortality prediction in patients aged 65 years and older (AUC = 0.888, 95% CI 0.856–0.921), followed by ISS (AUC = 0.867, 95% CI 0.831–0.903), whereas RTSc showed substantially lower performance (AUC = 0.687, 95% CI 0.644–0.730). The optimal cutoff values were RTSc ≤ 7.55, ISS ≥ 8, and TRISS ≤ 97%. Of the three scales, TRISS achieved the highest Youden index (0.701), indicating the best overall predictive performance. The results of pairwise AUC comparisons are presented in Table 8.

**Table 8:** Pairwise comparison of AUC values using DeLong’s test

Comparison	AUC of first scale	AUC of second scale	Difference in AUC	z	*p*-value
ISS vs. TRISS	0.867	0.888	-0.021	-2.095	0.036
ISS vs. RTSc	0.867	0.687	0.180	7.335	< 0.001
TRISS vs. RTSc	0.888	0.687	0.201	9.738	< 0.001

DeLong’s test demonstrated that TRISS performed significantly better than ISS (*p* = 0.036). Both ISS and TRISS were significantly superior to RTSc (ISS vs. RTSc: *p* < 0.001; TRISS vs. RTSc: *p* < 0.001).

## Discussion

This study compared three commonly used trauma scoring systems – RTSc, ISS, and TRISS – in a cohort of older trauma patients to identify the most appropriate retrospective reference tool for evaluating trauma center (TC) qualification criteria in geriatric patients. Several findings merit emphasis.

First, TRISS demonstrated the highest discriminatory ability for in-hospital mortality (AUC 0.888), followed by ISS (AUC 0.867), whereas RTSc performed substantially less well (AUC 0.687). Importantly, DeLong’s test showed that the difference between TRISS and ISS, although modest, was statistically significant (*p* = 0.036), and both scales were significantly superior to RTSc. Second, conventional physiological thresholds identified only a small proportion of older patients with severe injury. Third, all patients in the TC group 65 + had ISS ≥ 16, despite only 38.46% having RTSc < 4. Taken together, these findings indicate a substantial discordance between anatomical injury burden and physiological presentation in older trauma patients and raise concern that current TC qualification criteria may fail to identify a relevant proportion of geriatric patients with severe trauma.

In a previous study based on the same institutional cohort, we demonstrated that current TC qualification criteria may be inadequate for identifying severely injured geriatric patients [51]. The present analysis extends these findings by identifying an objective retrospective reference standard for evaluating such criteria. Its novelty lies not in re-demonstrating the prognostic value of TRISS per se, which has been reported previously, but in applying established trauma scoring systems to assess the adequacy of national TC qualification criteria in older adults within the still-evolving Polish trauma system. In this context, TRISS may serve as a robust retrospective benchmark for system-level evaluation, providing a methodological foundation for future multicenter validation and refinement of geriatric trauma triage criteria.

### RTSc scale

The performance of RTSc was limited in the present cohort. Although an RTSc < 4 has traditionally been considered a threshold indicating the need for TC care [9], only 0.43% of patients in *the trauma group 65 +* met this criterion. This observation supports previous suggestions that the conventional cutoff may be underestimated in older adults [8]. This proportion was lower than the percentage of patients meeting the national TC qualification criteria (0.60%), which previous national studies have also indicated may be overly restrictive [42]. Moreover, this subgroup had a very low mean TRISS-predicted probability of survival (13.28%).

Furthermore, among patients who met the national TC qualification criteria, only 38.46% had an RTSc < 4. This finding suggests that, in this subgroup, anatomical injury burden (reflected by higher ISS) may have had a greater influence on prognosis than physiological derangement alone.

RTSc is based on physiological parameters, including the Glasgow Coma Scale (GCS) and systolic blood pressure (SBP), whose values in older adults may differ from those typically observed in younger trauma populations. Studies on the pathophysiological characteristics of ageing suggest that the shock threshold accepted in younger adult populations may be insufficiently sensitive in older trauma patients [43].

In ROC analysis, the optimal RTSc cutoff for in-hospital mortality prediction was ≤ 7.55; however, sensitivity at this threshold remained low (38.20%), despite very high specificity (99.00%). In practical terms, this suggests that RTSc alone is unlikely to be sufficient as a reference standard for evaluating triage performance in older adults. In another study assessing RTSc for mortality prediction in geriatric trauma patients, the optimal cutoff was 6, with a sensitivity of 99% but considerably lower specificity of 62% [44]. More balanced sensitivity and specificity values were reported in another comparable study, in which the optimal RTSc cutoff was ≤ 7.108 (sensitivity 97%, specificity 80%) [8, 40].

### ISS scale

ISS performed substantially better than RTSc and showed good discriminatory ability for in-hospital mortality prediction, with an AUC of 0.867. In the present study, the optimal ISS cutoff was ≥ 8, with a sensitivity of 82.10% and a specificity of 84.80%. In another study of in-hospital mortality prediction in geriatric trauma patients, the optimal ISS cutoff was 13.5, with sensitivity of 84% but substantially lower specificity (62%) [44]. In a further comparable study, the best sensitivity and specificity were observed at an ISS cutoff of ≥ 16, which corresponds to the conventional threshold for severe injury, with a sensitivity of 91% and a specificity of 89% [8].

From a clinical and systems perspective, however, the more informative finding may be that all patients in *the TC group 65 +* had ISS ≥ 16, and the median ISS in this group was 38, indicating very high injury severity. In the broader *trauma group 65+*, patients with ISS ≥ 16 accounted for 2.25% of the cohort (*n* = 147), whereas only 39 patients fulfilled the national TC qualification criteria.

ISS is widely regarded as one of the best predictors of injury-based mortality [29] and is commonly used as a reference standard for assessing injury severity [45]. Although it is not suitable for real-time prehospital triage, because it is an anatomical score based on definitive diagnostic findings, ISS remains a valuable retrospective marker of injury severity and a useful benchmark for evaluating triage performance. However, like most general trauma scoring systems, it was developed for populations spanning all adult age groups, and its performance in the oldest injured patients remains uncertain [36, 46]. Moreover, as a purely anatomical scoring system, ISS does not incorporate physiological variables, age, or pre-existing comorbidities, all of which may substantially influence prognosis in geriatric trauma patients [36]. Accordingly, some studies have reported limited ability of ISS to predict mortality in patients aged > 65 years [12, 47].

### TRISS scale

TRISS showed the best overall discriminatory ability for predicting in-hospital mortality. In ROC analysis, the optimal TRISS cutoff for in-hospital mortality prediction in *the trauma group 65 +* was ≤ 97%, with a sensitivity of 85.40% and a specificity of 84.70%, indicating well-balanced predictive performance. This cutoff should be viewed as a retrospective benchmarking tool rather than a bedside triage threshold.

In another study evaluating TRISS in geriatric trauma patients, the optimal cutoff was ≤ 91.6%, with higher sensitivity (97%) and specificity (87%) [8]. Such differences between studies are not unexpected and likely reflect variations in patient populations, injury patterns, and case mix.

The principal strength of TRISS lies in its integration of anatomical injury severity, physiological status, and age, a combination recognized as a strong predictor of mortality in older adults [8, 44]. This combined structure likely explains its better performance than RTSc and its small but statistically significant advantage over ISS. Although the superiority of TRISS over ISS should be interpreted with caution, the present findings suggest that an integrated approach may be particularly informative in geriatric trauma, in which age substantially modifies risk.

In *the TC group 65+*, the mean TRISS-predicted probability of survival was 34.58%, whereas observed survival to hospital discharge was 48.72%. This suggests that TRISS underestimated actual survival in the subgroup with the greatest injury burden. A plausible explanation is that the original TRISS coefficients may not fully reflect contemporary trauma care or outcomes in very old patients. This interpretation should be treated with caution, also because of the small size of the TC subgroup. Reports indicating that trauma-related mortality in older adults may extend well beyond hospitalization, with substantial one-year mortality after injury, should also be considered [25].

Additional limitations of TRISS should also be acknowledged. First, the score uses physiological parameters recorded on hospital admission, which may differ from prehospital baseline values. Second, because TRISS incorporates the Glasgow Coma Scale, it may be difficult to calculate accurately in patients who are intubated or sedated before hospital arrival [36].

TRISS is not intended for real-time triage or trauma center activation because it relies on anatomical and physiological information that becomes available only after diagnostic workup and initial in-hospital assessment. Its utility therefore lies primarily in retrospective analysis, where it may serve as an objective reference standard for evaluating whether current triage and TC qualification criteria adequately identify older patients at high risk of death. This distinction is essential for interpreting the present findings.

TRISS is a widely accepted prognostic model [35, 48] that has been used in trauma outcomes research, benchmarking, and quality assessment [1, 5, 6, 33]. Previous studies in geriatric trauma populations have also shown that TRISS performs better than ISS, RTSc, and several other commonly used trauma scores in predicting mortality [8, 44]. This pattern is further supported by recent meta-analytic evidence, which found that the pooled discriminatory performance of TRISS (AUC 0.82) was higher than that of ISS (AUC 0.74) and GTOS (AUC 0.80) in older trauma patients [36]. In another study, ISS (AUC 0.807) also performed less well than TRISS (AUC 0.828), although the highest AUC in that analysis was achieved by NISS (AUC 0.850) [49].

It should also be noted that ISS remains more commonly reported in trauma registries and observational studies, partly because it is more readily available. Consequently, age-related prognostic effects may be insufficiently captured when trauma system performance is evaluated primarily on the basis of anatomical scores alone.

### Trauma center eligibility in older adults

The present findings suggest that the TC qualification criteria currently adopted in Poland may be overly restrictive for older trauma patients, as they appear to exclude a substantial proportion of geriatric patients with severe injuries. Using the undertriage estimate most commonly applied according to the Cribari method, based on the proportion of patients with ISS ≥ 16 [42, 50], the undertriage rate in the present cohort would be as high as 73.47%.

This figure exceeds rates reported in many international studies, in which fewer than half of older trauma patients are appropriately transferred to higher-level trauma care and the risk of inadequate triage increases with age [25]. It also markedly exceeds the undertriage threshold of < 5% considered acceptable for older adults by the American College of Surgeons Committee on Trauma (ACS-COT) [32].

The very low number of older patients who met the national TC qualification criteria over the four-year study period further reinforces concerns that the current criteria may be overly restrictive in this population. In contemporary trauma systems, timely access to advanced trauma care aims to reduce preventable trauma-related mortality and facilitate standardized management pathways [48]. Current research primarily focuses on prehospital criteria for triage to TCs and trauma team activation (TTA). Although contemporary studies have proposed age-adjusted modifications—such as increasing the systolic blood pressure threshold, adjusting GCS cutoffs, and incorporating the shock index—substantial heterogeneity in findings persists. Despite ongoing efforts to develop geriatric-specific triage systems, no clear consensus has yet been reached [51]. In this context, the selection of appropriate retrospective reference tools may represent a crucial step toward developing more consistent and effective geriatric triage criteria. Importantly, this study was not intended to propose a practical prehospital triage tool. These findings support evaluation of age-adjusted or geriatric-specific trauma triage approaches, highlighting the need for further research to define effective triage protocols for geriatric trauma patients.

## Limitations

This study has several limitations. First, its retrospective, single-center design limits the generalizability of the findings to other trauma systems and healthcare settings. In addition, because no national trauma registry currently exists in Poland, international comparisons and broader population-level validation remain limited. Consequently, ISS and TRISS values had to be estimated manually, which may have increased the risk of subjectivity and potential classification errors. Second, the small size of the TC subgroup may have reduced the precision of subgroup-based estimates and limited the strength of conclusions regarding TC qualification criteria. Third, trauma scores were calculated using physiological parameters recorded on admission, which may differ from prehospital measurements. Finally, although TRISS showed the best discrimination for in-hospital mortality, this finding should be interpreted in light of the fact that TRISS is a composite model incorporating ISS, RTSc, and age adjustment.

## Conclusion

Current national trauma center qualification criteria may not adequately identify older patients with severe trauma and may contribute to undertriage in this population. Among the evaluated scoring systems, TRISS showed the best discrimination for in-hospital mortality and appears to be the most appropriate retrospective reference standard for assessing the adequacy of trauma center qualification criteria in geriatric trauma patients. These findings support further multicenter evaluation and possible refinement of trauma center qualification criteria for older adults, with particular consideration of age-sensitive approaches to trauma triage.

## Data Availability

No datasets were generated or analysed during the current study.

## Abbreviations

TC: Trauma center
ED: Emergency department
AUROC: Area under the ROC curve
ICD: International classification of diseases
RR: Respiratory rate
SBP: Systolic blood pressure
GCS: Glasgow coma scale
Ps: Probability of survival
PPV: Positive predictive value
NPV: Negative predictive value
AUC: Area under the curve
